# Mortality risk and social network position in resident killer whales: sex differences and the importance of resource abundance

**DOI:** 10.1098/rspb.2017.1313

**Published:** 2017-10-25

**Authors:** S. Ellis, D. W. Franks, S. Nattrass, M. A. Cant, M. N. Weiss, D. Giles, K. C. Balcomb, D. P. Croft

**Affiliations:** 1 Centre for Research in Animal Behaviour, University of Exeter, Exeter EX4 4QG, UK; 2 Department of Biology, University of York, York YO10 5GE, UK; 3 Centre for Ecology and Conservation, University of Exeter in Cornwall, Penryn, Cornwall TR10 9FE, UK; 4 Center for Whale Research, 355 Smugglers Cove Road, Friday Harbor, WA 98250, USA

**Keywords:** *Orcinus orca*, social networks, life history, survival analysis, fitness, sociality

## Abstract

An individual's ecological environment affects their mortality risk, which in turn has fundamental consequences for life-history evolution. In many species, social relationships are likely to be an important component of an individual's environment, and therefore their mortality risk. Here, we examine the relationship between social position and mortality risk in resident killer whales (*Orcinus orca*) using over three decades of social and demographic data. We find that the social position of male, but not female, killer whales in their social unit predicts their mortality risk. More socially integrated males have a significantly lower risk of mortality than socially peripheral males, particularly in years of low prey abundance, suggesting that social position mediates access to resources. Male killer whales are larger and require more resources than females, increasing their vulnerability to starvation in years of low salmon abundance. More socially integrated males are likely to have better access to social information and food-sharing opportunities which may enhance their survival in years of low salmon abundance. Our results show that observable variation in the social environment is linked to variation in mortality risk, and highlight how sex differences in social effects on survival may be linked to sex differences in life-history evolution.

## 1. Introduction

An individual's mortality risk, their chance of dying at a given time, has fundamental evolutionary consequences [1]. Many aspects of life history have been shown to be linked to mortality risk such as age at first reproduction, parental care strategy and senescence [2]. Interestingly, males and females of the same species often have differing mortality risks due to their differing reproductive strategies [3]. For example, costly displays and intrasexual aggression increase the risk of male mortality in many mammal and bird species [4–6]. This has in turn been linked to life history; for example, higher rates of senescence have been reported in males of some polygynous species [7]. Understanding factors that govern the mortality risk of individuals has the potential to explain many important aspects of life history and behaviour, and why they vary between the sexes.

Social behaviour is important for many species and may affect individual mortality risk. In humans, for example, mortality risk has been linked to a variety of aspects of sociality (e.g. [8–10]). Social behaviours are usually direct, occurring between pairs or groups of individuals. However, an important feature of social behaviour is that these direct interactions form part of a complex network of interactions. An individual's position within this network, their social position, depends partly on their own interactions and partly on the interactions of others. An individual's social position will determine in part its access to resources and social information (information that can be learnt by observation of, or interaction with, other individuals [11–16]) and may therefore have implications for their mortality risk. Indeed, in several species social position has been linked to early life mortality risk [17–20]. However, the difficulty in collecting long-term dynamic social data means that the proximate mechanism by which sociality can affect mortality risk over the lifetime of individuals are largely unknown. To understand the ultimate processes driving the evolution of life-history strategies, and why these strategies may differ between the sexes, it is important to understand the link between social position and mortality risk.

Killer whales (*Orcinus orca*) are a particularly interesting species in which to study the relationship between sociality, sex, ecology and mortality risk. They are highly social: resident killer whales off the Pacific Northwest coast of the USA and Canada live in hierarchical societies [21], the social structure of which changes in different ecological conditions [22]. Social information is important to allow whales to find food, especially when resources are scarce [23], and family relationships also have important effects on the survival of individuals [24,25]. Male killer whales differ from females in their body mass [26], feeding habits [27] and likelihood of responding to social information [23]. In addition, resident killer whales have sexually divergent lifespans, with females who reach maturity predicted to live to 53, whereas males who reach maturity are unlikely to survive past 29 [25]. Killer whales are also one of only three mammals where females are known to have an evolutionary significant post-reproductive lifespan [28]. The importance of social relationships, their sexually divergent lifespans and their unusual life histories make killer whales a good species in which to study the relationship between sociality and mortality risk.

Here, we test the hypothesis that social position is linked to mortality risk in resident killer whales using 34 years of social and demographic data. Specifically, we (i) quantify the relationship between social position and survival in male and female resident killer whales and (ii) link this to resource abundance, to give an insight into the proximate mechanisms driving the relationship between sociality and mortality.

## 2. Methods

### (a) Study site

This study was conducted on the southern resident killer whales inhabiting the waters off the coast of Washington State, USA and British Colombia, Canada. The population has been studied by annual photographic census undertaken by Orca Survey since 1976. In the summer, the southern residents inhabit the area around the San Juan islands, feeding almost exclusively on Chinook salmon (*Oncorhynchus tshawytscha*) [29,30]. The abundance of salmon in a given year has a significant impact on the mortality risk of resident killer whales [29,30]. The southern residents are a closed population of 71–98 individuals (between 1976 and 2010), with no social or genetic exchange with other sympatric killer whale populations [31,32]. Resident killer whales inhabit a hierarchical society. The smallest unit is the matriline consisting of the offspring and grand offspring of a female [21] and neither sex disperse, staying in close association with their matriline their whole life [21]. Higher levels include pod, community and clans, sharing broadly similar dialects and movement patterns [21].

### (b) Data

Social associations were taken from the annual photographic census of whales between 1976 and 2010. Whales photographed within three body lengths (or within three body lengths of another group member) were considered to be part of the same group (electronic supplementary material, S1). Association groups were defined within an encounter (an observation of a group/groups of whales; see electronic supplementary material, S1). Group sizes ranged from single individuals to aggregations of 24 whales, with a mean of 2.5 whales per group. Each whale was observed a mean (±standard error) of 31.3 ± 0.67 times per year. Pairs of whales were observed together from 1 to 149 times per year with a mean (±standard deviation) of five (±7.9) observations per dyad. We use these association groups to construct a social network based on the ‘gambit of the group’ paradigm with the strength of association between two individuals calculated based on simple ratio indices [33,34] (see electronic supplementary material, S1). Whales in close association have the opportunity to hunt together and share food [27]. Our association measure therefore indicates the frequency with which individuals hunt, travel and socialize together. For the years 1990–2010, all data from a given year were used to calculate a single social network for each year. Between 1976 and 1989, there was a comparatively lower sampling effort and photographic data were sparser than in later years. To ensure that we had sufficient data to reliably infer social structure [33], in these early years we combined data into 2-year sampling periods to construct the social networks. The southern resident killer whales are monitored when they are inshore during the salmon runs in the summer. Over winter, they are usually further offshore and not observed. Most deaths occur over the winter. A whale is considered to have died in a given sampling period if they are not observed in the next summer (electronic supplementary material, S1).

Salmon abundance in the Pacific Northwest varies greatly between years based on the El Niño–Southern Oscillation and, more recently, fishing activity [35,36]. We use the salmon index calculated from test fisheries (begun in 1979) taken in the summer range of the southern resident killer whales as an estimate of salmon abundance ([37]; also used in [22,23]). The absence of data means that data from 1976 to 1978 are not included in the analysis including salmon abundance. Approximately half of all whale deaths occur in years with the lowest quartile of salmon abundance (34 of 65 deaths). For analysis, we classify the years with this lowest quartile of abundance as low salmon years, and the other years as high salmon years.

### (c) Social communities

Resident killer whales preferentially associate with a small number of individuals [21]. This is reflected in the distribution of social association strength in the community [38], which is characterized by some strong bonds and many weaker bonds (mean ± s.d. social differentiation, *S* =1.09 ± 0.18; electronic supplementary material, S1). The biologically relevant social position for a whale with regard to survival and ecology is most likely to be within these smaller groups of important associates, rather than over the whole population. In this study, we therefore concentrate on an individual's social position within these networks of preferential associates.

**Figure 1.**
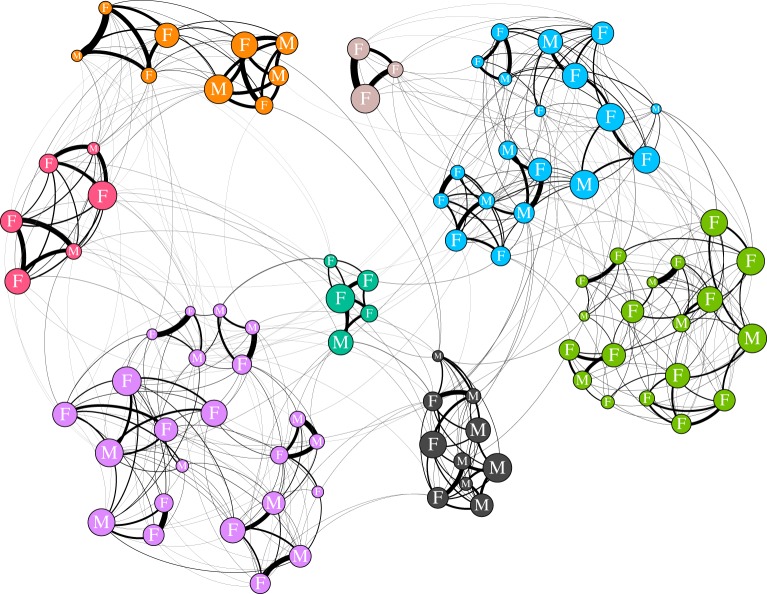
An example annual social network for the southern resident killer whale population (1996). Node colour shows community membership. Node size is scaled by a whale's normalized rank indirect centrality (within-community closeness). Node labels represent sex: M = male, F = female. Edge width is based on the simple rate index between two whales. Network visualized in Gephi.

We use community analysis to allow the groups of preferential associations to emerge from the patterns of social interaction. Communities were detected in each weighted annual social structure using a random walk algorithm [39]. Communities were defined independently in each network (example: figure 1). These communities are a good representation of the social structure of the population as a high proportion of social associations are within, rather than between, communities: modularity was in the range 0.63–0.84. The killer whale population is significantly more modular than random (1000 data-stream permutations [see below], *p* < 0.001 in all years; median modularity of permuted datasets was 0.13 [interquartile range = 0.07–0.23]). The assignment of individuals to communities is robust. Individuals assigned to the same community in the original dataset are consistently grouped together in 1000 bootstrapped replicates: *r*_com_ = 0.81 ± 0.01 (mean ± standard deviation; electronic supplementary material, S1; [40,41]). As a measure of confidence in our calculated within-community network structure, we estimated the correlation between our observed association indices and the ‘true’ association indices [38]. Our within-community association indices are a good (greater than 0.4, [38]) reflection of the ‘true’ association pattern: mean (±standard deviation) annual *r* = 0.75 ± 0.09 (electronic supplementary material, S1; [38]).

Annually, the population contained a median of six large (greater than five whales; see below) communities (range 5–9). Each large community contained an average of 11 ± 5.9 (mean ± standard deviation) whales. Communities tend to consist of a matriline, extended matriline or group of regularly associating matrilines. Throughout this study we use ‘community’ to refer to the small units derived from the social associations, rather than the larger separation between the northern and southern resident populations, which have also sometimes been referred to as communities [21].

### (d) Quantifying social position

Social centrality is perhaps the most commonly used measure of social position. In essence, social centrality describes how well connected an individual is to others in their social system [33]. Social centrality therefore describes how well positioned an individual is to receive, for example, information, resources or disease from other members of their society [11–15]. In practice, an individual's likelihood of accessing resources or information held by other individuals will depend on two factors: (i) their direct connections to others, governed partly by their own social decisions, and (ii) their indirect connections to others in their community, which partly depend on the social choices of others [42]. We use two measures to capture the indirect and direct aspects of social centrality: closeness and degree. Closeness is the inverse of the average path length: the (weighted) number of steps from one individual to all others in the community. A high closeness indicates small number of weighted steps to all other individuals in the community and therefore a high social centrality. Degree is a simple unweighted count of a whale's associates. This can be seen as a measure of the number of social partners an individual has and hence their potential access to information and resources held by other members of the community [33]. Closeness depends on an individual's social position in the network and the indirect interactions, and the strength of the interactions, it has with other individuals. In contrast, degree depends only on an individual's direct associations. In this study, we are interested in an individual's social position within their network of close associates, their community. We therefore calculated centrality measures (community closeness and community degree) within an individual's local social community. To account for differing sizes of communities and annual populations, both measures were normalized for analysis. Measures were not calculated on communities with fewer than five whales, as patterns of social connection are limited in such small groups. Additionally, as closeness is highly skewed, it was also ranked within the network before normalization. These two measures quantify different but related aspects of social centrality. As would be expected, the measures are correlated, but the correlation is weak and highly variable (see electronic supplementary material, S2).

### (e) Statistical analysis

We use survival analysis to investigate the social factors affecting the mortality of whales over their observed lifetime. Survival analysis describes an individual's probability of surviving beyond a specified time [43]. We analyse mortality risk using Cox proportion hazard (Cox PH) models which describe how the instantaneous risk (hereafter risk) of death occurring at a given time is affected by covariates. We use extended Cox PH models to investigate how a whale's probability of survival is affected by their position within the social network, which can change with time. For each Cox PH model we report the estimated hazard ratio (Haz.: calculated as the exponential of the model coefficient). A hazard ratio of exactly 1 indicates no differences in risk of mortality as the variable changes; a hazard ratio of less than 1, therefore, indicates a decreasing risk of mortality with a higher value of the variable. The hazard ratio is directly equivalent to increases (or decreases) in mortality risk per unit of variable i.e. a hazard ratio of 0.25 indicates a 75% decrease in the risk of mortality per unit of variable. Generalized linear mixed-effect models (GLMMs) were used for analyses not investigating survival. All GLMMs have a binomial error structure with whale identity as a random effect.

To account for the inherent autocorrelation in network data, we used permutations to produce a null distribution to compare to the observed data. Unless indicated otherwise, analyses presented here are based within social communities. For this within-community analysis, we used 10 000 within-community node permutations to produce the null models (see electronic supplementary material, S1). When analysing at the population level (rather than the within-community level), we used 10 000 data-stream permutations to produce the null models (indicated by superscript ^d.s.^ by the test name; see electronic supplementary material, S1). Additionally within each permutation, for whales of unknown sex we assigned a sex at random with 1000 imputations per null model. The mean network statistic of these 1000 sex imputations was used as the network statistic for that iteration of the broader network permutation. Permuted null models were created for all Cox PH models [15], and GLMMs analysing networked data. For each permutation of the data the model (Cox PH or GLMM) is applied to the randomized data. These permuted data are used to create a null distribution of the test statistic which is then compared to the observed value [33]. For permuted analyses, reported *p* values indicate the number of times the simulated test statistic was greater than or equal to the observed test statistic (sample included in the numerator and denominator [44]). For analysis not including network statistics, permutations were not used (indicated by the superscript ^n.p^ by the test name). All analyses were performed in R using the igraph, ggplot2, lme4 and survival packages.

## 3. Results

### (a) Sex, centrality and survival

**Figure 2.**
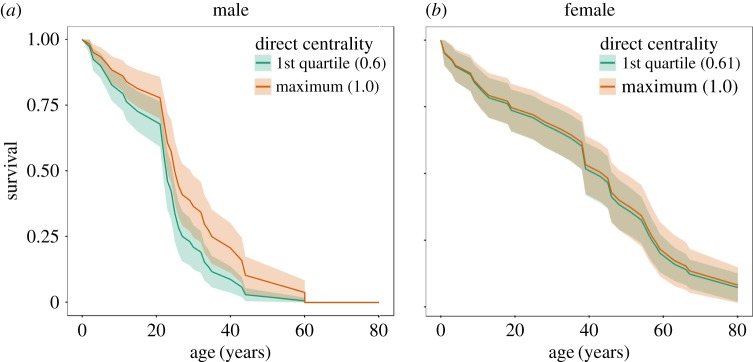
The survival of male (*a*) and female (*b*) killer whales based on their network centrality predicted by Cox PH survival models. The two curves represent the predicted survival of whales with the first quartile and maximum direct centrality (degree) of whales observed in the study. The survival of male whales is significantly related to their centrality within their social community (Haz. = 0.21 ± 0.14; Cox PH, *z* = −1.95, *e* = 41, *p* = 0.0009), whereas female survival is not (Haz. = 0.85 ± 0.66; Cox PH, *z* = −0.005, *e* = 40, *p* = 0.112).

Within social communities, both direct and indirect social centrality significantly relates to the survival probability of male, but not female killer whales. Males with a higher community closeness (a measure of indirect centrality) have a significantly lower mortality risk than whales with a lower community closeness (Haz. = 0.33 ± 0.2; Cox PH, *z* = −1.67, events (*e*) = 41, *p* = 0.0366; figure 2*a*). In contrast, the survival of female killer whales is unrelated to their closeness centrality (Haz. = 1.06 ± 0.72; Cox PH, *z* = 0.03, *e* = 40, *p* = 0.6924; figure 2*b*). Similarly, males associating with a larger number of individuals in their community (within-community degree) have a significantly lower mortality risk than individuals with fewer associates (Haz. = 0.21 ± 0.14; Cox PH, *z* = −1.95, *e* = 41, *p* = 0.0009). In contrast, female within-community degree does not significantly influence survival (Haz. = 0.85 ± 0.66; Cox PH, *z* = −0.005, *e* = 40, *p* = 0.112). The effect of social centrality on survival is not simply an artefact of social group size: network community size does not influence the survival of either sex (male, Haz. = 0.99 ± 0.02, Cox PH^d.s.^, *z* = −0.42, *n* = 860, *e* = 41, *p* = 0.8529; female, Haz. = 0.406 ± 0.26, *z* = −1.76, *n* = 1410, *e* = 39, *p* = 0.7999).

### (b) Centrality, survival and salmon

**Figure 3.**
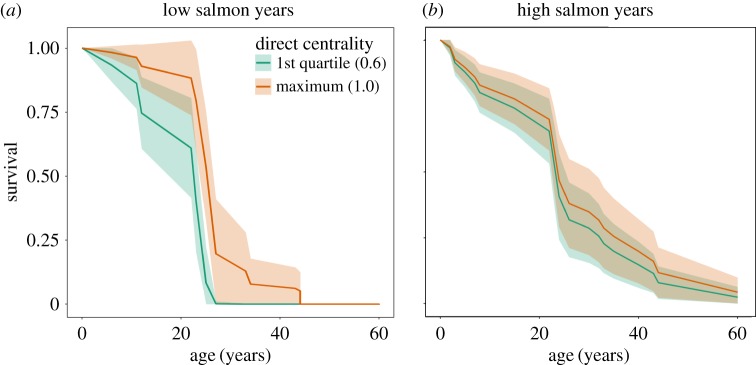
The survival of male killer whales in years of low (*a*) and high (*b*) salmon abundance. Lines show the survival of males with different direct social centrality as predicted by Cox PH models. Social centrality significantly relates to survival in years of low salmon abundance (Haz. = 0.002 ± 0.006; Cox PH, *z* = −3.24, *e* = 41, *p* = 0.0001) but not in years of high salmon abundance (Haz. = 0.641 ± 0.72; Cox PH, *z* = −0.24, *e* = 41, *p* = 0.06).

We find that both male (Haz. = 0.10 ± 0.09; Cox PH^n.p.^, *z* = −2.7, *n* = 825, *p* = 0.001) and female (Haz. = 0.03 ± 0.03; Cox PH^n.p.^, *z* = −4.16, *n* = 1335, *p* < 0.001) survival is significantly related to salmon abundance. Salmon abundance has an important influence on the relationship between social position and male survival; in years of low salmon abundance, males with high social centrality have a lower mortality risk (community degree: Haz. = 0.002 ± 0.006; Cox PH, *z* = −3.24, *e* = 41, *p* = 0.0001; community closeness: Haz. = 0.02 ± 0.038; Cox PH, *z* = −2.28, *e* = 41, *p* = 0.014; figure 3). In contrast, in years of higher salmon abundance, social centrality does not significantly relate to male mortality risk (community degree: Haz. = 0.641 ± 0.72; Cox PH, *z* = −0.24, *e* = 41, *p* = 0.06; community closeness: Haz. = 1.03 ± 0.92, Cox PH, *z* = −0.10, *e* = 41, *p* = 0.4706; figure 3). The survival of females is not significantly related to their social centrality in years of either low salmon abundance (community degree: Haz. = 0.36 ± 0.42; Cox PH, *z* = −0.72, *e* = 39, *p* = 0.215; community closeness: Haz. = 0.51 ± 0.52; Cox PH, *z* = −0.53, *e* = 39, *p* = 0.3046) or high salmon abundance (community degree: Haz. = 0.95 ± 1.2, Cox PH, *z* = 0.09, *e* = 39, *p* = 0.1168; community closeness: Haz. = 1.54 ± 1.77, Cox PH, *z* = 0.17, *e* = 39, *p* = 0.4706).

Interestingly, males do not have a higher degree in years of low salmon abundance (GLMM: *β* = 0.14, *z* = 0.46, *n* = 742, *p* = 0.2449). This suggests that even though degree is important for survival in years when salmon are scarce, males do not appear to increase their direct centrality by associating with more individuals.

## 4. Discussion

Our results show that the survival of male, but not female, southern resident killer whales is significantly related to their social position in their local community. Males in the most central social positions (indirect centrality) have one-third of the mortality risk of those in the least central social positions. This is linked to salmon abundance: social position is only related to male survival in years of low salmon abundance.

Social position could affect the survival of males by mediating their access to resources via a number of mechanisms. For example, social information about the location of salmon has been shown to be important for resident killer whales who often follow post-reproductive females during collective movement, particularly during years of low salmon abundance [23]. A central social position may also increase a male's likelihood of receiving food from other whales. Resident killer whales, particularly females, often share fish that they catch with close associates [27]. An individual with more associates may be more likely to be the recipient of these food-sharing events, increasing their food intake, and in turn increasing their probability of survival. Other social factors may affect both social position and access to resources directly. Dominance, for example, could mediate both an individual's social position (e.g. [45–47]) and access to resources (e.g. [48,49]).

Social mechanisms will be most important when resources are scarce in years of low salmon abundance. Male killer whales may be more reliant than females on socially mediated resources because male resident killer whales are substantially larger than females: adult female southern resident killer whales are estimated to weigh up to 3338 kg, whereas adult males are estimated to weigh up to 4434 kg [26]. Males are estimated to require a 25% higher energetic intake to maintain this body size [26,50]. This higher energetic requirement of males will make them more vulnerable to starvation (e.g. [51–53]). A higher reliance on social information and food sharing may explain why the survival of males, but not females, depends on their position within the social structure.

Given the importance of the number of associates to the survival of male killer whales, it is interesting that they do not appear to increase their number of associates in response to low salmon abundance. There are possible behavioural and social explanations for this lack of social flexibility. Behaviourally, animals are time-limited, and face a trade-off between behaviours, including social and foraging behaviours [54]. In times of low resource availability, foraging pressures may reduce time available for social interactions, preventing males from increasing their social centrality, even when it would increase their probability of survival. The presence of such a trade-off is supported by observations of the behaviour of resident killer whales which have a lower rate of association in years of low salmon abundance [55] and a less interconnected social network [22], suggesting that they are engaging in less social behaviour. Competition for resources could also prevent males increasing their social centrality in years of low salmon abundance. Smaller group sizes in low resource conditions have been observed in a range of species (e.g. [56,57]). In a similar process, individuals may choose not to associate with a given male in times of low salmon abundance to reduce resource competition for themselves or their closer relatives. These non-exclusive mechanisms may explain why males appear unable to increase their social centrality in times of low salmon abundance.

Sociality can decrease an animal's mortality risk in a variety of ways. By living in groups animals can, for example, decrease their risk of predation or increase their foraging efficiency [58]. Recently the importance of heterogeneity in sociality has been highlighted, and a body of work shows that animals within a population can have a wide variety of social positions and social roles [59]. The heterogeneity of social bonds can have important fitness consequences. In several species, an individual's social bonds and affiliation (rather than social position) have been shown to affect their survival [60–62], reproductive success [18,63,64] and the survival of their offspring [17,18]. In addition, infant survival has been linked to social position in savannah baboons (*Papio cynocephalus*) [20]. However, to our knowledge, this is the first demonstration that social position is related to the survival of individuals in a complex society over their whole lifetime. In addition, we show that both the direct and indirect components of social position have survival implications and link these social effects to ecological conditions. Linking social structure and survival has many important evolutionary and ecological consequences [65]. For example, if fitness varies for different individuals within a group, we expect selection for individuals to move to these favourable social positions by changing their social strategy. However, an individual's position in the broader social structure is dependent on the behaviours and decisions of others in the population. Selection cannot, therefore, act directly on social position: it is difficult to imagine behavioural choices an individual could make to increase their indirect centrality. This highlights how behavioural phenotypes within a group interact to produce the fitness outcomes for the group members [66]. For male killer whales this means that their mortality risk partly depends on social factors outside their control which may contribute to their higher mortality rate in comparison to females. This may have fundamental life-history consequences, for example, by selecting for investment in reproduction in early life, and leading to an earlier onset of senescence [1,4].

In this study, we have shown that the survival of male resident killer whales is related to their position within a complex social system, especially in years of low resource abundance. Most models of social and life-history evolution assume a relatively constant and homogeneous social environment. In this study, we show that this may be an underestimation of the complexity of the evolutionary consequences of sociality, and that the heterogeneity and dynamics of social systems can have fundamental fitness consequences.

## Supplementary Material

Supplementary Material 1

## Supplementary Material

Supplementary Material 2

## Data accessibility

Data to replicate the statistical tests are available from the Dryad Digital Repository: https://doi.org/10.5061/dryad.654sm [67]. Requests for access to raw data can be directed to the authors or the Center for Whale Research (www.whaleresearch.com).
