# Supplementary material for: Mortality risk and social network position in resident killer whales: sex differences and the importance of resource abundance
Source: Proc Biol Sci. 2017 Oct 25;284(1865):20171313. doi: 10.1098/rspb.2017.1313 (PMC5666093; doi:10.1098/rspb.2017.1313)
Supplement: Supplementary Material 1 [file rspb20171313supp1.docx]

1. The Relationship between community degree and community closeness

There is a signficant relationship between community closeness and community degree (GLMM Est=4.26, z=16.079, n=2067, p<0.01). However, as can be seen from this figure this correlation is neither close nor consistent.


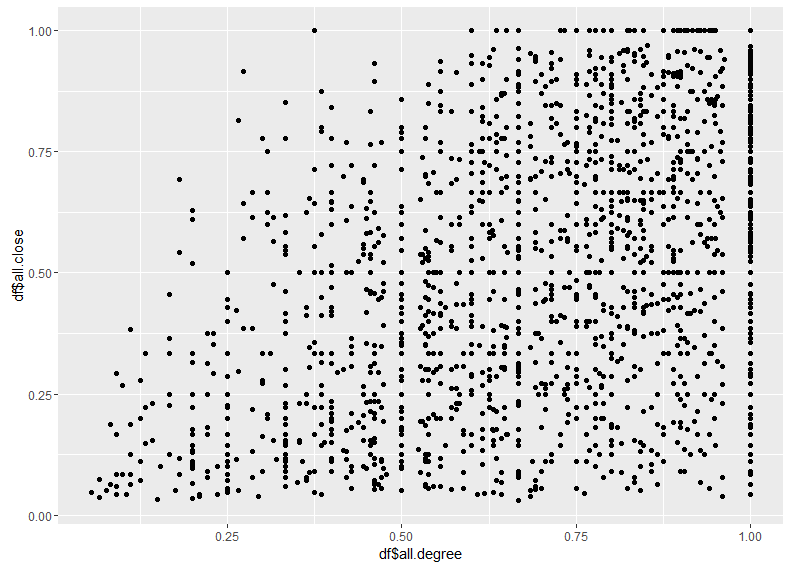


Community Closeness

Community Degree

1. Relationship between observations and survival

*Males*


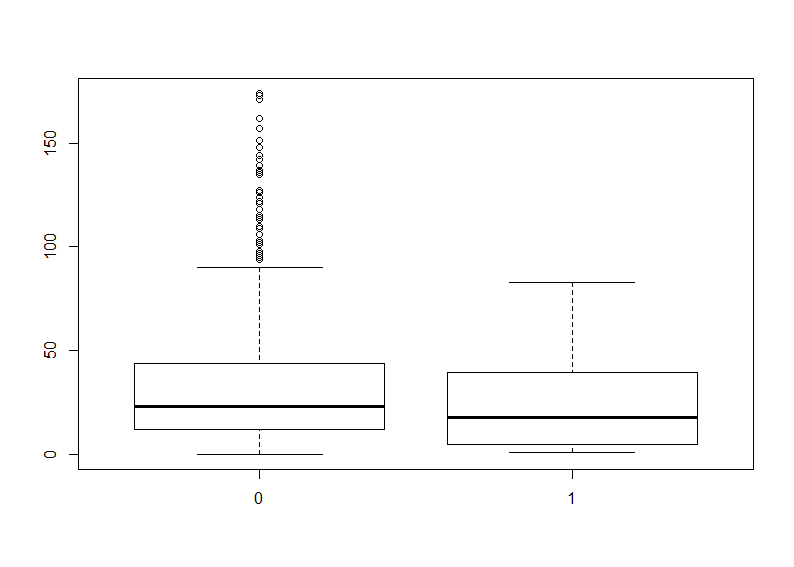


Survive

Die

Whale fate in year *x*

Number of Observation in year *x*

There is no significant difference between number of observations of males in years that they die and years in which they survive (Est= -0.009, z=-1.3, n= 860, p= 0.21).

*Females*


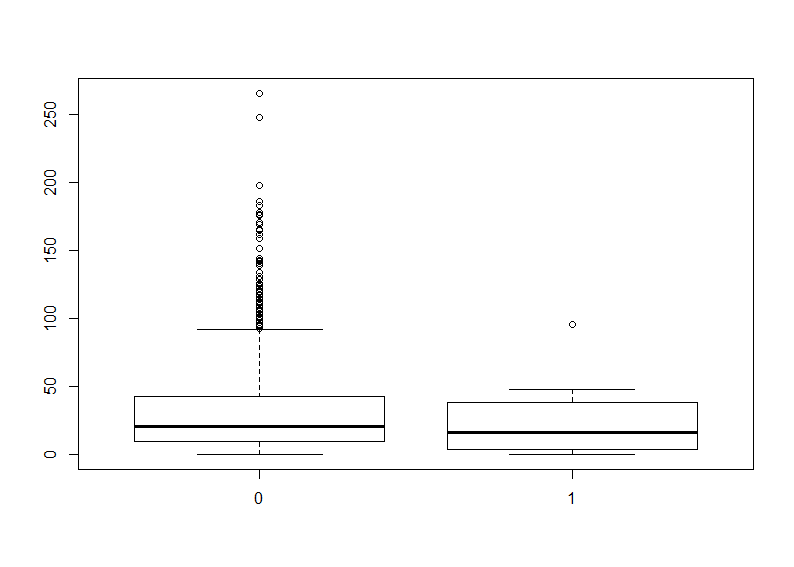


Survive

Die

Whale fate in year *x*

Number of Observation in year *x*

There is no significant difference between number of observations of females in years that they die and years in which they survive (Est= -0.0.13, z=-1.8, n= 1410, p= 0.08).
