# Supplementary material for: Mortality risk and social network position in resident killer whales: sex differences and the importance of resource abundance
Source: Proc Biol Sci. 2017 Oct 25;284(1865):20171313. doi: 10.1098/rspb.2017.1313 (PMC5666093; doi:10.1098/rspb.2017.1313)
Supplement: Supplementary Material 2 [file rspb20171313supp2.docx]

**Data collection**

Annually between June and August Orca Survey performed regular *ad hoc* boat surveys in the feeding grounds of the resident killer whales of Washington State, USA and British Colombia, Canada. Whales were encountered on an average 43±4.45 (mean ± SE) survey days per year. During a survey whales encountered together were photographed with high quality cameras. Whales were individually identified based on unique marking patterns by experienced observers [1]. Marks are unique and distinctive and identification errors by experienced observers are very rare.

We use the gambit of the group paradigm: assuming individuals in close spatial association are interacting [2,3], which is a common approach in the study of animal sociality [4]. We define whales as within the same group if they are within three body lengths of one another. When encountered in groups whales were most commonly travelling, resting or socialising and occasionally foraging, though most commonly hunting was undertaken outside of the three body length distance [5]. Whales travelling and socialising have shallow (less than 3m) and short dives (less than 30 seconds) [6] and surface together or in very quick succession. Patterns of association above water are therefore likely to be representative of below water association. In addition we employ a chain rule [7] so individuals within three body lengths of an associate are still considered to be part of the same group. An average of 1043.29±142.24 encounters were observed per year.

**Social network construction**

We used our observed association groups to construct annual networks of social association. The strength of the association between two individuals was estimated based on simple ratio indices [4,8]. The simple ratio index for assessing the strength of social affiliation between two individuals and is given as the probability of each individual being observed [4,8]. Networks were calculated annually between 1990 and 2010, and for two years of association between 1976 and 1989 to control for the comparatively lower sampling effort in the earlier years of the study (mean annual observations: 1976-1990=228, 1990-2010=1053).

We calculated how the distribution of our calculated association indices reflect the distribution of the ‘true’ observed associations as a measure of confidence in our calculated network structure [9]. Social differentiation in the observed data (approximated using the methods described in [9]) was high: within-community *S* = 1.25 ±0.09; table S1. Within-communities the calculated association indices accurately reflect the estimated ‘true’ association pattern (*r*= 0.75 ± 0.09; table S1).

**Statistical analysis**

Individuals within social networks are, by definition, not independent, violating the assumption of most standard statistical tests [10]. To control for this it is necessary to create null model expectations based on permutations of the data [4,8,10].

Our analysis is based on measuring effects within communities. Using data stream permutations [4,11] in this case is inappropriate because all randomly constructed networks will have a different community structure. Data stream randomisations permute the groups individuals are associated in (in our case the observed association groups) [11]. Within a data-stream permutation, a given individual will occur in communities with very different structural properties, and will therefore have a very high variability in their within-community network position. Using data-stream randomisations will therefore overestimate the variability an individual experiences in their community network structure and will therefore overestimate the significance of any variable during analysis. Conversely using data-stream permutations, while preserving community structure will have insufficient variability to produce a useful null model. We therefore use node-based randomisations to construct the null models for within-community analysis**.** Node-based randomisations are based on permuting the properties of the nodes in a network to provide a randomised network with the same structural properties as the original observed network [8,7]. In our analysis individuals are assigned a permuted position within their community, the network properties are then calculated, which is used as the null model. Although node-based randomisations will not compensate for group size biases [11,12], it does allow us to preserve community structure which means it provides a more representative null model for this analysis. When testing the effect of community size on survival we used data stream permutations (10 000) as these analysis did not rely on consistent community structure (superscript ^d.s.^)

In both cases, from each permuted network the test statistic (*z*) is calculated, and compared to that from the real data. The reported p value is the proportion of times the test statistic from 10 000 permutations is larger than that from the real data. All p values testing network effects are based on this process.

**References**

1. Bigg, M. A., Olesiuk, P. F., Ellis, G. M., Ford, J. K. B. & Balcomb, K. C. 1990 Social organization and genealogy of resident killer whales (Orcinus orca) in the coastal waters of British Columbia and Washington State. *Reports Int. Whal. Comm.* **SI 12**, 383–405.

2. Whitehead, H. & Dufault, S. 1999 Techniques for analyzing vertebrate social structure using identified individuals: review and recommendations. *Adv. Study Behav.* **28**, 33–74. (doi:10.1016/S0065-3454(08)60215-6)

3. Franks, D. W., Ruxton, G. D. & James, R. 2010 Sampling animal association networks with the gambit of the group. *Behav. Ecol. Sociobiol.* **64**, 493–503. (doi:10.1007/s00265-009-0865-8)

4. Farine, D. R. & Whitehead, H. 2015 Constructing, conducting and interpreting animal social network analysis. *J. Anim. Ecol.* **84**, 1144–1163. (doi:10.1111/1365-2656.12418)

5. Ford, J. K. B. & Ellis, G. M. 2006 Selective foraging by fish-eating killer whales *Orcinus orca* in British Columbia. *Mar. Ecol. Prog. Ser.* **316**, 185–199. (doi:10.3354/meps316185)

6. Wright, B. M., Ford, J. K. B., Ellis, G. M., Deecke, V. B., Shapiro, A. D., Battaile, B. C. & Trites, A. W. 2017 Fine-scale foraging movements by fish-eating killer whales (Orcinus orca) relate to the vertical distributions and escape responses of salmonid prey (*Oncorhynchus* spp.). *Mov. Ecol.* **5**, 3. (doi:10.1186/s40462-017-0094-0)

7. Croft, D. P., James, R. & Krause, J. 2008 *Exploring animal social networks.* 1st edn. New Jersey, USA: Princeton University Press.

8. Whitehead, H. 2008 *Analyzing Animal Societies: Quantative methods for vertebrate social analysis*. Chicago: University of Chicago Press.

9. Whitehead, H. 2008 Precision and power in the analysis of social structure using associations. *Anim. Behav.* **75**, 1093–1099. (doi:10.1016/j.anbehav.2007.08.022)

10. Croft, D. P., Madden, J. R., Franks, D. W. & James, R. 2011 Hypothesis testing in animal social networks. *Trends Ecol. Evol.* **26**, 502–7. (doi:10.1016/j.tree.2011.05.012)

11. Bejder, L., Fletcher, D. & BrÄger, S. 1998 A method for testing association patterns of social animals. *Anim. Behav.* **56**, 719–725. (doi:10.1006/anbe.1998.0802)

12. Farine, D. R. 2014 Measuring phenotypic assortment in animal social networks: Weighted associations are more robust than binary edges. *Anim. Behav.* **89**, 141–153. (doi:10.1016/j.anbehav.2014.01.001)

13. Shizuka, D. & Farine, D. R. 2016 Measuring the robustness of network community structure using assortativity. *Anim. Behav.* **112**, 237–246. (doi:10.1016/j.anbehav.2015.12.007)

Table S1: Social differentiation (*S)* and how will this represents the calculated association indices (*r*), calculated using the methods described in [9]. A value of *S* near of greater than 1indicate a population where individuals have highly differentiated social relationships. *r* is limited to between 0 and 1. *r*=1 suggests a near perfect representation of the ‘true’ association patterns in the calculated association indices. Values *r* greater than 0.4 are considered to show the calculated indices are a ‘fair’ representation of the true structure [8,9]. Values of *r* greater than 0.8 are considered to be a ‘good’ representation of the data [8,9]. Community assortivity robustness, r_com_, represents the how robust the assignment of a pair of individuals into the same community is given social structure and sampling [13]. All values are well above the 0.5 threshold suggested for a robust assessment of community structure [13]

| Year | Within Community S | Within-Community r | Community robustness (r_com_) |
| --- | --- | --- | --- |
| 1976/77 | 1.11 | 0.71 |  |
| 1978/79 | 1.26 | 0.61 |  |
| 1980/81 | 1.10 | 0.63 |  |
| 1982/83 | 1.15 | 0.60 |  |
| 1984/85 | 1.24 | 0.86 |  |
| 1986/87 | 1.29 | 0.76 |  |
| 1988/89 | 1.19 | 0.92 |  |
| 1990 | 1.38 | 0.70 |  |
| 1991 | 1.26 | 0.79 |  |
| 1992 | 1.27 | 0.77 |  |
| 1993 | 1.34 | 0.64 |  |
| 1994 | 1.37 | 0.63 |  |
| 1995 | 1.37 | 0.66 |  |
| 1996 | 1.28 | 0.75 |  |
| 1997 | 1.18 | 0.82 |  |
| 1998 | 1.06 | 0.77 |  |
| 1999 | 1.27 | 0.73 |  |
| 2000 | 1.15 | 0.87 |  |
| 2001 | 1.23 | 0.85 |  |
| 2002 | 1.21 | 0.85 |  |
| 2003 | 1.30 | 0.78 |  |
| 2004 | 1.36 | 0.75 |  |
| 2005 | 1.38 | 0.72 |  |
| 2006 | 1.19 | 0.81 |  |
| 2007 | 126 | 0.79 |  |
| 2008 | 1.38 | 0.60 |  |
| 2009 | 1.1 | 0.85 |  |
| 2010 | 1.19 | 0.74 |  |
| **Mean (± std. dev.)** | **1.25 (±0.09)** | **0.75 (±0.09)** |  |
